# Exercise-Induced Blood Lactate Increase Does Not Change Red Blood Cell Deformability in Cyclists

**DOI:** 10.1371/journal.pone.0071219

**Published:** 2013-08-05

**Authors:** Michael J. Simmonds, Philippe Connes, Surendran Sabapathy

**Affiliations:** 1 Heart Foundation Research Centre, Griffith Health Institute, Griffith University, Gold Coast, Australia; 2 UMR Inserm U665, Universite des Antilles et de la Guyane, Pointe-a-Pitre, Guadeloupe; 3 Universite des Antilles et de la Guyane, Laboratoire ACTES (EA 3596), Departement de Physiologie, Pointe-a-Pitre, France; 4 Laboratory of Excellence GR-Ex «The red cell: from genesis to death», PRES Sorbonne Paris Cité, Paris, France; Universidad Europea de Madrid, Spain

## Abstract

**Background:**

The effect of exercise-induced lactate production on red blood cell deformability and other blood rheological changes is controversial, given heavy-exercise induces biochemical processes (e.g., oxidative stress) known to perturb haemorheology. The aim of the present study was to examine the haemorheological response to a short-duration cycling protocol designed to increase blood lactate concentration, but of duration insufficient to induce significant oxidative stress.

**Methods:**

Male cyclists and triathletes (*n* = 6; 27±7 yr; body mass index: 23.7±3.0 kg/m^2^; peak oxygen uptake 4.02±0.51 L/min) performed unloaded (0 W), moderate-intensity, and heavy-intensity cycling. Blood was sampled at rest and during the final minute of each cycling bout. Blood chemistry, blood viscosity, red blood cell aggregation and red blood cell deformability were measured.

**Results:**

Blood lactate concentration increased significantly during heavy-intensity cycling, when compared with all other conditions. Methaemoglobin fraction did not change during any exercise bout when compared with rest. Blood viscosity at native haematocrit increased during heavy-intensity cycling at higher-shear rates when compared with rest, unloaded and moderate-intensity cycling. Heavy-intensity exercise increased the amplitude of red blood cell aggregation in native haematocrit samples when compared with all other conditions. Red blood cell deformability was not changed by exercise.

**Conclusion:**

Acute exercise perturbs haemorheology in an intensity dose-response fashion; however, many of the haemorheological effects appear to be secondary to haemoconcentration, rather than increased lactate concentration.

## Introduction

Initiation of exercise redistributes fluids away from the plasma compartment, increasing the packed cell volume of blood and relative concentration of plasma proteins, leading to elevated blood and plasma viscosities [1]. Exercise-induced alterations to the red blood cell (RBC), including increased RBC aggregation and decreased RBC deformability, directly increase blood viscosity [2]. Given that blood flow and oxygen/nutrient delivery may be mediated by changes in viscosity (Poiseuille equation), haemorheological changes are postulated to influence exercise efforts that are heavily reliant upon oxidative metabolism [3].

Exercise intensity profoundly determines the physiological responses to physical activity. The so called ‘anaerobic threshold’ demarks moderate- from heavy-intensity exercise, and represents the lowest work rate at which the clearance of lactate is surpassed by the rate of lactate appearance in the blood [4]. Changes in blood chemistry which occur at the anaerobic threshold are thought to significantly alter haemorheology [1], [3]. Decreased blood pH, for example, has been consistently demonstrated to increase the rigidity of RBC [5], [6], which in-turn increases higher-shear blood viscosity [7]. Localised hypoxia during exercise above the anaerobic threshold is associated with lactate accumulation, though whether lactate directly alters haemorheology remains incompletely resolved. *In vitro* incubation with lactate significantly increases the rigidity of RBC [6], [8]. In contrast, exercise-induced increases of blood lactate concentration (*in vivo*) have not always been associated with a concomitant decrease in RBC deformability [9], despite RBC fragility being reported to increase in tandem with increased lactate concentration [8]. These conflicting results have been interpreted to suggest that there may be a potential training status effect [10], whereby RBC deformability of well-conditioned individuals may paradoxically increase in the presence of lactate. A confounding factor among the studies investigating the effects of exercise-induced lactate production on haemorheology is the prolonged duration of the exercise bouts utilised: prolonged heavy-intensity exercise induces other factors (e.g., oxidative stress) known to impact upon haemorheology [11], [12].

The aim of the present study was to investigate whether blood lactate accumulation during heavy-intensity exercise influences RBC deformability, specifically during a short-duration cycling bout not expected to induce oxidative stress. It was hypothesised that blood lactate produced during short-duration heavy-intensity exercise would be accompanied by a decrease in RBC deformability. A secondary aim was to characterise the exercise-intensity versus haemorheology relationship using well-defined exercise-intensity domains based on the anaerobic threshold.

## Methods

### Subjects

Six healthy male cyclists and triathletes volunteered to participate in the present study after providing written informed consent. Subjects were considered trained if they were cycling at least 200 km⋅wk^−1^ for at least 24 months. Subjects were non-smokers and not suffering from any known cardiovascular, pulmonary, or metabolic diseases. Each subject performed two exercise tests that were scheduled at a regular time in the early morning, with each test separated by at least 48 h. The subjects were instructed to refrain from intense physical activity and to abstain from consuming caffeine and alcohol for at least 24 h prior to visiting the laboratory. The experimental procedures were reviewed and approved by the Bond University Human Research Ethics Committee.

### Pre-experimental Protocol

Subjects were familiarised with the experimental procedures and equipment, and preferred setup (i.e., height and position measurements of the seat and handlebars) of the cycle ergometer (Lode Excalibur Sport V2.0, Groningen, The Netherlands) was recorded and applied for all subsequent exercise tests. The familiarisation session was also used to determine each subject’s preferred pedalling cadence (ranged between 80–110 rev·min^−1^) to be used during subsequent tests. All exercise tests were performed on the same ergometer and subjects were instructed to maintain their preferred pedalling cadence throughout each test, without leaving the saddle.

### Determination of VO_2_peak and the Gas-exchange Threshold

Peak exercise and gas exchange threshold (as a non-invasive marker of the anaerobic threshold) values were determined during an incremental cycling test performed to volitional fatigue. Following 4 min of cycling at 90 W, the work rate increased by 15 W every 30 s until the subject reached the point at which they were unable to maintain a pedalling cadence within 10 rev·min^−1^ of their preferred cadence despite strong verbal encouragement. Gas-exchange was measured breath-by-breath using open-circuit spirometry (Ultima CardiO_2_, Medical Graphics Corp., St Paul, MN) during the test. The oxygen and carbon dioxide analysers were calibrated before each test with precision reference gases, while calibration of the pneumotachograph was performed using a 3-L calibration syringe (Hans Rudolph Inc., Kansas City, USA). During the incremental exercise test, cardiac rhythm was monitored using a 12-lead ECG (Cardio Perfect, Welch Allyn Inc., Skaneateles Falls, USA) and brachial artery blood pressure was measured and recorded every 3 min. Peak VO_2_ was determined as the average of the highest values measured over two consecutive 30-s intervals. The gas-exchange threshold (T_ge_) was determined using the simplified V-slope method [13].

### Experimental Cycling Protocol

Participants returned to the laboratory on a separate day to perform a multiple-staged submaximal exercise bout. Upon arrival at the laboratory, an intravenous catheter was inserted into a prominent forearm vein, typically in the antecubital region. The intravenous line was flushed at regular intervals during exercise with 1–2 mL of 0.9% saline solution.

The multiple-staged exercise protocol involved the subjects cycling continuously for 24 min. The first 6 min was performed at 0 W (unloaded), after which a moderate-intensity workload, corresponding to 80% of the power output attained at the T_ge_, was applied without warning. The subjects continued moderate-intensity cycling for 6 min before the workload was removed for a further 6 min of unloaded cycling. The final exercise stage comprising 6 min of heavy-intensity cycling at a power output equal to 40% of the difference between the power attained at the gas exchange threshold and VO_2_peak (i.e., delta40), was then applied without warning. Breath-by-breath VO_2_ and heart rate were measured during the entire exercise session, as described for the incremental exercise test.

### Blood Sampling and Immediate Analyses

Prior to each blood sample collection, a small volume of blood was drawn from the intravenous catheter and immediately discarded to ensure the line was free of saline. Six mL of whole blood was collected into a sterile 10 mL syringe before exercise (rest), and during the final minute of each exercise stage. Bicarbonate concentration, pH, and methaemoglobin were determined immediately by a co-oximeter (ABL80 FLEX, Radiometer Medical ApS, Denmark) from the whole blood samples, while blood lactate (La^−^) concentration was immediately quantified using an automated lactate analyser (Lactate Pro LT-1710, ARKRAY, Japan). An 80-µL aliquot of whole blood was then drawn from each sample into glass capillary tubes and haematocrit determined using the microhaematocrit method, after 5 min of high-speed centrifugation (10,000 *g*). The remainder of the blood samples were immediately transferred into collecting tubes containing ethylenediaminetetraacetic acid (EDTA, 1.8 mg·ml^−1^) and stored at 37°C for haemorheological analysis, which was performed and completed within 4 h of collection, as recommended [14].

### Whole Blood Viscosity

Viscosity of blood (ηb) at native or 0.4 L/L haematocrit was measured at 37°C and shear rates of 75–1500 s^−1^ using a rotational cone-plate viscometer (0.5 DVII+ with CPE40 spindle, Brookfield Engineering Labs, Middleboro, MA).

### Determination of RBC Deformability

RBC deformability was determined at 37±1°C using laser-diffractometry. Briefly, 7 µL of whole blood from an EDTA tube was diluted in 700 µL of medium solution (5.5% Polyvinylpyrrolidone, mol·L^−1^ = 360,000, dissolved in 1 mol·L^−1^ PBS, osmolality = 300 mosmol·kg^−1^). The diluted blood (600 µL) was transferred to a test kit loaded into an ektacytometer (Rheoscan-D, Sewon Meditech Inc., Korea) for analysis; this ektacytometer has limited internal heating capacity, thus blood suspensions and microchannels were incubated at 37°C until immediately prior to analysis. In the ektacytometer, the sample was subjected to a range of shear stresses (0–25 Pa) by aspiration across a microchannel (∼200 µm), while a laser was directed through the channel. The laser-diffraction pattern from RBC suspensions was captured at 2 Hz by an integrated digital camera. The resultant images were analysed by fitting an ellipse to the diffraction pattern, and an RBC elongation index (EI) calculated for the different shear stress applied using the following equation: EI = (A – B)/(A+B), where A is the length of the major axis of the ellipse, and B is the length of the minor axis of the ellipse. Measurements were performed in duplicate, and the average EI values were subsequently fit with a non-linear regression (Prism, GraphPad Software Inc, Release 5.0, USA) to identify two parameters that describe RBC deformability: SS_1/2_, the shear stress required for half of maximal deformation, and; EI_max_, the maximum elongation index (deformability) at infinite shear stress [15].

### Determination of RBC Aggregation

Aggregation of RBC suspended in plasma at native and adjusted to 0.4 L/L haematocrit were determined using 35 uL samples of each suspension with a computerised aggregometer system (Myrenne Aggregometer, Myrenne GmbH, Roetgen, Germany). The aggregometer system is not temperature controlled, thus it was operated in an environmental chamber maintained at 37±1°C. Three dimensionless indices of RBC aggregation were determined for each suspension: i) M_0_, the extent of aggregation at stasis by integrating light transmission through the blood sample during 10 s following an abrupt cessation of high shear (600 s^−1^); ii) M_1_, the extent of aggregation at very low shear (3 s^−1^) also measured via light integration during 10 s following cessation of high shear; iii) AI_120_, the extent of aggregation at stasis by integrating light transmission through the blood sample during 120 s following an abrupt cessation of high shear (600 s^−1^). Measurements were performed in duplicate and the average values are reported.

### Data Analyses

Results are reported as means ± standard deviation. Normality of the data was tested using the D’Agostino & Pearson omnibus normality test (GraphPad Software Inc, Release 5.0, USA). Data for each sampling method were compared using a one-way ANOVA with repeated measures to determine whether significant differences in the means existed (SPSS Inc, Release 19.0, USA). Tukey’s adjustments were used when appropriate to determine differences between time points. Eta-squared (η^2^) was calculated to determine the mean difference effect for each dependent variable. Relationships between blood lactate concentration and RBC deformability were assessed using parametric correlation analyses: the delta change in dependent variables (e.g., lactate concentration; EI_max_, etc) was calculated for each exercise condition, when compared with rest (e.g., heavy vs rest), and the Pearson’s product moment and significance were investigated. Significance was determined at an alpha level of 0.05.

## Results

Physical characteristics and peak exercise values obtained during the incremental exercise test are presented in Table 1. Based on the power output measured at T_ge_ and VO_2_peak, the moderate-intensity cycling bout was performed at 193±17 W (i.e., 0.8×T_ge_) and the heavy-intensity cycling bout was performed at 306±19 W (i.e., delta40).

**Table 1:** Physical characteristics and exercise values obtained during incremental cycling.

Age, yr	27±7
Body mass, kg	79±8
Height, cm	183±8
VO_2_peak, L·min^−1^	4.02±0.51
VO_2_peak, mL·kg^−1^·min^−1^	52±8
Power at T_ge_, W	241±21
Power at RCT, W	327±27
Peak power, W	404±38

Data are mean ± standard deviation. VO_2_peak: peak oxygen uptake. T_ge_: gas exchange threshold. RCT: respiratory compensation threshold.

During moderate-intensity cycling, VO_2_ increased in a classic monoexponential manner, from 0.88±0.22 L·min^−1^ during unloaded cycling, to a steady-state value (2.58±0.25 L·min^−1^) within 2–3 min after the workload was applied. The baseline VO_2_ (0.93±0.22 L·min^−1^) during unloaded cycling immediately prior to heavy-intensity cycling was not different to that observed immediately prior to moderate-intensity cycling; however, steady-state VO_2_ was not attained during the heavy-intensity cycling bout, initially increasing to 3.50±0.36 L·min^−1^ at 3 min and further increasing to 3.80±0.31 L·min^−1^ at 6 min of exercise, indicative of the VO_2_ slow component during this stage of exercise.

**Figure 1:**
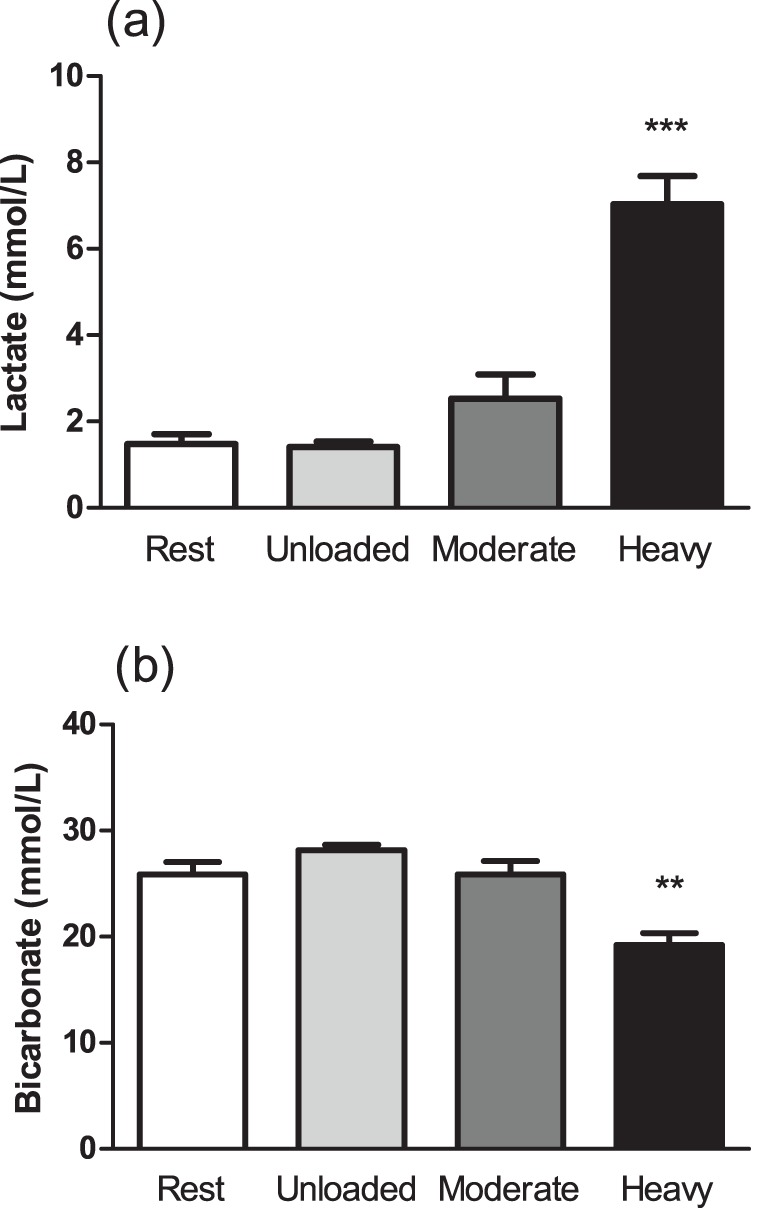
Blood lactate (a) and bicarbonate (b) concentration at rest and following 6-min unloaded, moderate-intensity, and heavy-intensity cycling. Data are mean ± standard error. ***, heavy significantly different, p<0.001. **, heavy significantly different, p<0.01.

Blood lactate and bicarbonate concentrations at rest and during the sixth minute of unloaded, moderate, and heavy exercise are illustrated in Figure 1. Blood lactate concentration after 6 min of unloaded cycling was not significantly different when compared with rest (1.5±0.5 mmol·L^−1^). Furthermore, blood lactate concentration was not significantly changed by moderate-intensity cycling; immediately prior to the heavy-intensity bout, blood lactate concentration remained not significantly different from rest (1.9±0.8 mmol·L^−1^). Heavy-intensity cycling significantly increased blood lactate concentration when compared with all prior sampling periods (Figure 1a; p<0.001; η^2^ = 0.82). Blood bicarbonate concentration demonstrated the inverse trend to that observed for lactate, where the only sampling period that was significantly different to rest (and moderate-intensity cycling) was observed after 6 min of heavy-intensity cycling (Figure 1b; p<0.01; η^2^ = 0.64). Blood pH during unloaded (7.34±0.02) and moderate-intensity (7.32±0.03) cycling were not different when compared with rest (7.38±0.04); however, heavy-intensity cycling resulted in a significant decrease in blood pH (7.26±0.04; p<0.01; η^2^ = 0.61).

**Figure 2:**
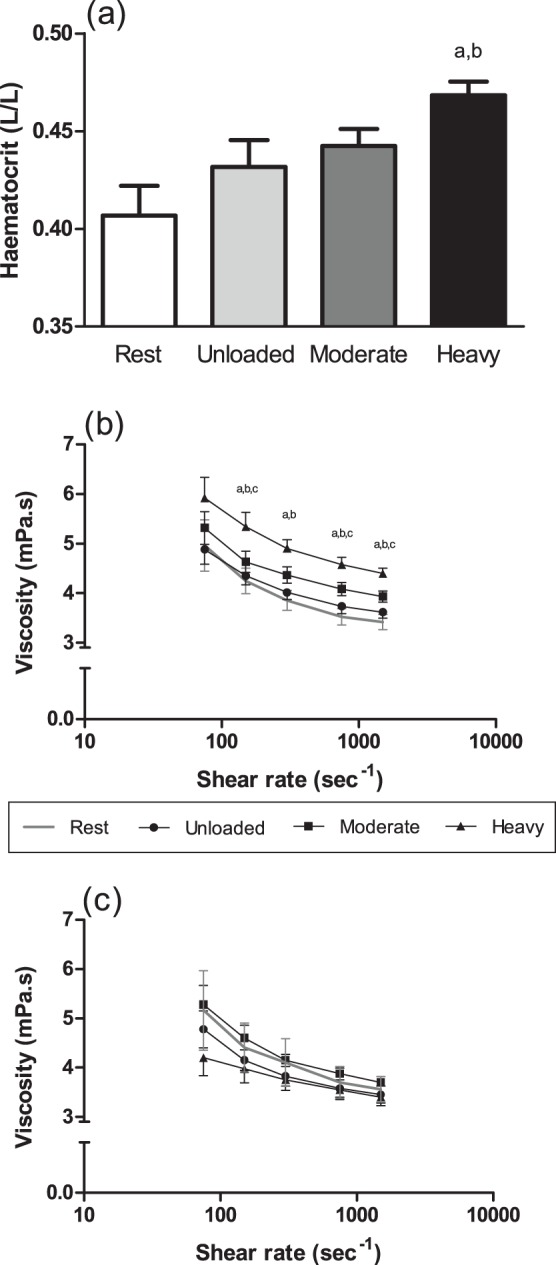
Haematocrit (a) and viscosity of RBC suspensions at native haematocrit (b) and adjusted to 0.4 L/L haematocrit (c) for rest and after 6-min unloaded, moderate-intensity, and heavy-intensity cycling. Data are mean ± standard error. ^a^, heavy significantly different to rest. ^b^, heavy significantly different to unloaded. ^c^, heavy significantly different to moderate.

Methaemoglobin was not altered due to 6 min cycling, irrespective of exercise intensity (Table 2). Haematocrit increased significantly (p<0.01; η^2^ = 0.42) during moderate and heavy-intensity cycling, when compared with rest (0.41±1.5 L/L). No significant difference was observed between haematocrit during moderate-intensity cycling, and unloaded cycling; however, when compared with unloaded cycling, heavy-intensity cycling increased haematocrit (p<0.05; Figure 2a).

**Table 2:** Methaemoglobin and aggregation of red blood cells in plasma at native and standardised haematocrit.

	Rest	Unloaded	Moderate	Heavy
Methaemoglobin, %	1.0±0.1	1.0±0.2	0.9±0.3	1.1±0.2
Native haematocrit				
M_0_, a.u.	5.0±1.1	5.2±0.8	6.5±0.9	7.9±0.6a,b
M_1_, a.u.	7.4±0.9	6.8±0.8	7.0±0.5	6.8±0.5
AI_120_, a.u.	71.7±2.0	74.5±1.5	78.4±1.5^a^	82.0±0.8a,b
Standardised haematocrit				
M_0_, a.u.	4.0±0.7	4.4±0.5	5.0±1.3	6.2±0.8
M_1_, a.u.	6.0±0.7	6.9±0.7	8.2±1.1	9.0±0.6
AI_120_, a.u.	72.6±1.6	74.0±1.0	73.9±2.0	75.9±1.2

M_0_: red blood cell (RBC) aggregation after 10 s at stasis. M_1_: RBC aggregation after 10 s at 3 sec^−1^. AI_120_: RBC aggregation after 120 s at stasis. a.u.: arbitrary units. Data are mean ± standard error.

a significantly different to unloaded.

b significantly different to rest.

Typical non-Newtonian shear-thinning behaviour was observed for η_b_ at native haematocrit (Figure 2b) and at 0.4 L/L haematocrit (Figure 2c). Moderate-intensity cycling did not significantly change η_b_ for either suspension, when compared with unloaded cycling. Heavy-intensity cycling significantly increased native haematocrit η_b_ at all measured shear rates ≥150 s^−1^, when compared with rest and unloaded cycling (all p<0.01; η^2^: range 0.45–0.63). Moreover, native haematocrit η_b_ was significantly higher at 150, 750, and 1500 s^−1^ during heavy-intensity cycling, when compared with moderate-intensity cycling. When blood was adjusted to 0.4 L/L haematocrit, however, no significant differences in η_b_ were observed (all p>0.05; η^2^: range 0.11–0.18).

**Figure 3:**
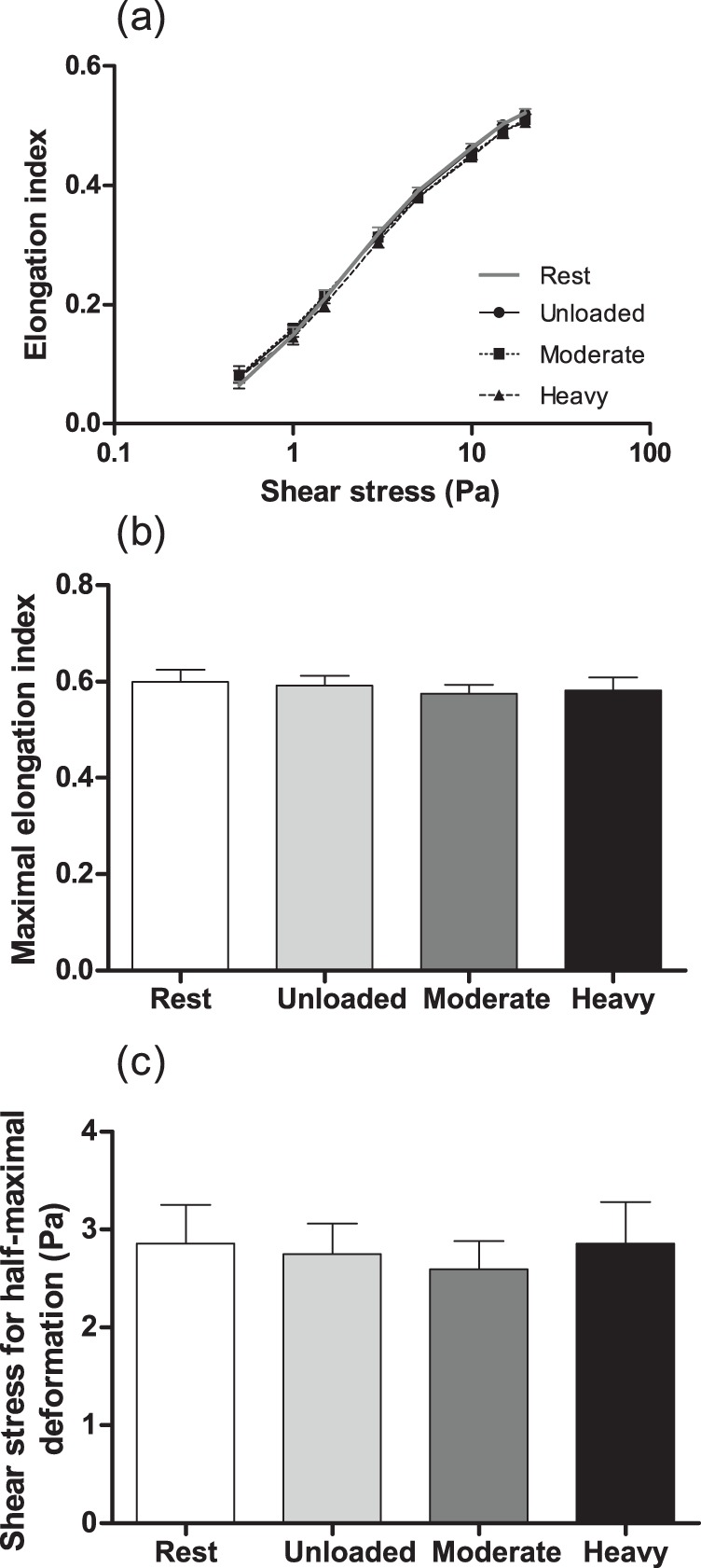
Red blood cell deformability measured at shear stresses between 0.5 and 20 Pa (a); maximal elongation index at infinite shear stress (EI_max_; b), and; shear stress required for half-maximal deformation (SS_1/2_; c). Data collected at rest, and after 6-min unloaded, moderate-intensity, and heavy-intensity cycling. Data are mean ± standard error.

The relationship between RBC deformability and shear stress was typically sigmoidal and EI increased with the rise in shear stress (Figure 3a). No significant difference was observed for RBC deformability between any exercise condition and rest. Moreover, exercise intensity did not significantly change EI at any shear stress at all measured shear stress (p>0.05; η^2^: range 0.00–0.14) or the curve-fit parameters describing RBC deformability – EI_max_ (p = 0.44; η^2^ = 0.11) and SS1/2 (p = 0.80; η^2^ = 0.05) – as illustrated in Figures 3b and 3c. Changes in blood lactate concentration, as well as blood pH, during exercise were not significantly associated with changes in RBC deformability parameters (*r* generally <0.1; all p>0.05).

The effect of short-term exercise on RBC aggregation is presented in Table 2. For RBC suspended in plasma at native haematocrit, moderate-intensity cycling did not alter RBC aggregation indices when measured over 10 s (i.e., M_0_ and M_1_); however, when RBC aggregation was determined over 120 s, AI_120_ was increased during moderate-intensity cycling, when compared with rest (p<0.05). Heavy-intensity cycling had a more profound effect on RBC aggregation at native haematocrit. Heavy-intensity cycling significantly increased RBC aggregation indices over 10-s (M_0_, p<0.05; η^2^ = 0.31) and 120 s (AI_120_, p = 0.001; η^2^ = 0.57), when compared with rest and unloaded cycling. For blood samples adjusted to a standardised haematocrit, no significant difference in RBC aggregation was detected (all p>0.05; η^2^: range 0.03–0.18).

## Discussion

The salient findings of the present study were that during heavy-intensity exercise which induced ∼8 mmol/L increase in blood lactate concentration and increased haematocrit by ∼15%: i) RBC deformability was unchanged; ii) η_b_ increased during heavy-intensity exercise; iii) RBC aggregation significantly increased. Collectively, the effect of acute exercise on haemorheology is related to the intensity at which exercise is performed, and many of the haemorheological effects appear to be secondary to haemoconcentration.

The results of the present study do not support our primary hypothesis − RBC deformability was not changed due to increased blood lactate concentration during a short-duration exercise bout. Oostenbrug et al., [16] reported a very small decrease in RBC deformability at a discrete shear stress (e.g., −1.6% only at 3.0 Pa) following a prolonged heavy-intensity exercise bout. More impressively, Yalcin et al., [17] reported that RBC deformability decreased by ∼7% following a 30-s “all-out” cycling effort; however, significant differences were only reported at a single shear stress (1.58 Pa). In contrast, SS_1/2_, but not EI_max_, measured using capillary samples collected at the earlobe, was recently reported to be decreased during 30-min treadmill running at 70% VO_2_peak [9]. These incongruent findings may reflect differences in exercise mode (e.g., cycling vs. running), or perhaps the population investigated. Connes et al., [18] reported decreased RBC rigidity (inverse of deformability) following maximal exercise only among subjects with normal haemoglobin saturation; RBC rigidity did not change in those subjects presenting with desaturation.

It has been postulated from *in vitro* studies that lactate could alter RBC deformability due to cell shrinkage, particularly when blood lactate concentration >4.0 mmol/L [19]. The present study clearly demonstrated, however, that increased blood lactate concentration of ∼8 mmol/L *in vivo* was not accompanied by decreased RBC deformability during 6 min of heavy-intensity cycling. The increase in blood lactate concentration can be solely attributed to the heavy-intensity exercise period, given that moderate-intensity cycling bout did not significantly alter blood lactate concentration from resting levels. Furthermore, there were no significant correlations between the changes in RBC deformability and blood lactate concentration in the present study. A potential explanation for the incongruent findings of the present and previous studies, is that studies which reported significant correlations between blood lactate concentration and altered RBC deformability implemented longer duration (e.g., 20–60 min) exercise bouts, whereas we used exercise of much shorter duration. During heavy-intensity exercise of a longer duration, other biochemical/cellular processes, such as oxidative stress, may develop and directly alter cellular rigidity [11], [20]. In the present study, the heavy-intensity cycling bout increased blood lactate concentration, but did not alter methaemoglobin, a marker of RBC oxidative stress [12]. The lack of change in methaemoglobin, coupled with the unaltered RBC deformability during exercise (RBC deformability is highly sensitive to oxidative stress [12]), suggests that short-duration heavy-intensity cycling did not induce *significant* oxidative stress. We are careful, however, not to definitively exclude the possibility of oxidative stress occurring in the present study, given the complexity of this phenomenon and that we only measured a single marker. In light of these findings, previous reports of altered RBC deformability during exercise may not necessarily be directly related to the production of lactate *per se*, but may be secondary to other biochemical processes.

It is puzzling that the decreased blood pH accompanying lactate production did not result in altered RBC deformability, given that the subsequent RBC dehydration triggered by activation of the volume- and pH-dependent K^+^/Cl^−^ cotransport channels should theoretically result in decreased RBC deformability [8]. Any potentially “adverse” effects of lactate and pH on RBC deformability may have been balanced by the reported beneficial effects of exercise-induced nitric oxide production on RBC deformability. Suhr et al., [21] demonstrated that improved RBC deformability following 60-min of moderate-intensity exercise occurred concomitantly with increased phosphorylation of RBC nitric oxide synthase and increased intracellular nitric oxide concentration. On the other hand, a two day intense training camp was associated with a down-regulation of RBC nitric oxide content and nitric oxide synthase activity [22]. Increased RBC nitric oxide production *in vitro* is associated with improved RBC deformability [23], confirming the importance of the role of nitric oxide in regulating this RBC rheological property. Based on these findings, it appears that an interaction between exercise intensity and duration is fundamental to the activation of RBC nitric oxide synthase, and thus, subsequent improvements in RBC deformability; this hypothesis requires further investigation.

The secondary aim of the present study was to characterise the effects of exercise-intensity on haemorheology using well-defined exercise intensity domains. In the present study, unloaded and moderate-intensity cycling did not alter η_b_, whereas heavy-intensity cycling significantly increased η_b_. The ∼25–30% increase in η_b_ due to heavy-intensity cycling agrees with values reported for blood sampled in the post-exercise period [1]. Exercise-induced increases in η_b_ are principally mediated by elevated haematocrit and plasma viscosity [3]. During short-term exercise, haemoconcentration may be observed in the order of 10–15%, secondary to fluid shifts away from the plasma [24], while plasma viscosity may also increase [25]. The effects of haemoconcentration are complex, given that elevated haematocrit improves oxygen transport capacity while also increasing η_b_ [26]. Compounding the effect of increased haematocrit on η_b_, fluid shifts result in a relative increase in plasma protein and macromolecule concentration – each directly increase plasma and blood viscosities. Increased η_b_ during exercise is associated with increased vascular shear stress, which is required for systemic adaptations in vascular function following exercise training [27]. Consequently, under conditions of increased metabolic demand, the determinants (e.g., haemoconcentration, hyperviscosity) that promote blood delivery and those which hinder it are not so clear. Nevertheless, it appears that during short-duration exercise, increased η_b_ may be only observed during heavy-intensity cycling as a consequence of haemoconcentration.

Red blood cell aggregation in 10-s (M_0_) significantly increased due to heavy-intensity exercise in the present study, but was not changed during unloaded or moderate-intensity cycling. On the other hand, RBC aggregation in 120-s (AI_120_) was significantly increased for both moderate- and heavy-intensity cycling bouts. Previous studies reported unchanged [28] and increased [29] RBC aggregation during exercise. Aggregation of RBC is determined by intrinsic (e.g., cell surface charge) and extrinsic (e.g., plasma fibrinogen concentration, haematocrit, shear rate, etc) factors [2]. During exercise, it is possible that a relative increase in plasma fibrinogen would accompany haemoconcentration. Since fibrinogen is a primary determinant of RBC aggregation, a relative increase in plasma fibrinogen concentration could explain, in part, the exercise-induced increase in RBC aggregation. However, haemoconcentration also increases the opportunity for cell-to-cell contact, and can largely explain the elevated RBC aggregation during exercise in the present study, given that aggregation was not different for suspensions with standardised haematocrit. Aggregation indices measured in native haematocrit samples are meaningful, as these represent the dynamic changes observed during exercise.

The functional consequence of elevated RBC aggregation is the axial migration of RBC away from vessel walls, reducing the RBC and oxygen content within various vascular routes (i.e., plasma skimming) [30], [31]. The resultant cell-poor layer at the vessel wall is associated with decreased flow resistance; however, the eventual need for RBC to disaggregate prior to entering smaller capillary networks induces an energy cost that may mitigate the benefits observed in larger vessels (for review, see [32]). Given the role of shear stress in promoting vasodilatory agents, it is plausible that RBC aggregation may serve as a means for regulating local blood flow in metabolically active tissue, whereby flow-resistance is minimised in free-flowing and larger vessels, thereby “reserving” the energy cost and resultant vascular shear stress for the microcirculation. Future studies investigating the role of RBC aggregation during exercise may yield further insights.

Collectively, the results of the present study do not support the hypothesis that blood lactate accumulation during short-duration heavy-intensity exercise decreases RBC deformability. Moreover, we were able to demonstrate that short-duration cycling bouts: i) did not alter RBC deformability; ii) increased blood viscosity, but only during heavy-intensity efforts; iii) increased RBC aggregation during moderate- (AI_120_) and heavy-intensity (M_0_, AI_120_) efforts. While increased blood viscosity and RBC aggregation during heavy-intensity exercise most likely occurs secondary to haemoconcentration, it is possible that this phenomenon provides a benefit for the regulation of localised oxygen and nutrient delivery.
